# Online media exposure and weight and fitness management app use correlate with disordered eating symptoms: evidence from the mainland of China

**DOI:** 10.1186/s40337-022-00577-y

**Published:** 2022-04-25

**Authors:** Lei Guo, Lian Gu, Yihua Peng, Yiming Gao, Li Mei, Qing Kang, Chen Chen, Yanran Hu, Wenyan Xu, Jue Chen

**Affiliations:** 1 Department of Clinical Psychology, Shanghai Mental Health Center, Shanghai Jiao Tong University School of Medicine, No. 600 South Wanping Road, Shanghai, 200030 People’s Republic of China; 2 School of Social Development and Public Policy, Fudan University, No. 600 Guoquan Road, Shanghai, 200433 People’s Republic of China

**Keywords:** Online media, Weight and fitness management app, Disordered eating, Chinese mainland, Young adults

## Abstract

**Background:**

The relationship between online media exposure and disordered eating symptoms has been reported in western regions. Though the prevalence of eating disorders and access to the Internet increased substantially in recent years, relevant evidence is rare in mainland China. This study aims to evaluate the association between online media exposure or weight and fitness management app use and disordered eating symptoms in Chinese mainland young adults, and the mediation effect of disordered eating cognition.

**Methods:**

353 Chinese mainland female and 142 male young adults completed online questionnaires consisting of demographic information, Eating Disorder Examination-Questionnaire 6.0 (EDE-Q 6.0), and items relating to online media exposure and weight and fitness management app use. Through correlation analysis, the relationship between online media exposure or weight and fitness management app use and disordered eating symptoms was examined, separately by sex. The mediation effect of disordered eating cognition on the relationship between online media exposure or weight and fitness management app use on disordered eating behaviors was investigated with two moderated mediation models.

**Results:**

Young female adults in the Chinese mainland presented higher disordered eating symptoms and were more engaged in online media and weight and fitness management app use than males. Online media exposure and weight and fitness management app use showed a significant correlation with disordered eating behaviors in males and females. Disordered eating cognition mediated the relationship between online media exposure or weight and fitness management app use and disordered eating behaviors. This effect was significantly higher in females.

**Conclusion:**

Online media exposure and weight and fitness management app use play a crucial role in the generation of disordered eating symptoms in Chinese mainland young adults, especially in females. The mediation analysis suggested the importance of prevention and intervention of disordered eating cognition. Monitoring and scientific guidance of online media are necessary.

**Supplementary Information:**

The online version contains supplementary material available at 10.1186/s40337-022-00577-y.

## Plain summary

Online media and weight and fitness management apps are becoming more than common in daily lives. Many studies reported that the peer pressure and the ideal body conveyed by online media, as well as calculating calories with weight and fitness management apps, may induce disordered eating behaviors. In this study, we examined this association in Chinese mainland young adults. Our results suggested that in young adults in the mainland of China, spending more time on online media and using weight and fitness management apps were closely correlated to disordered eating cognition and behaviors. Exposure to online media or using weight and fitness management apps induce disordered eating behaviors through evoking disordered eating cognition. What’s more, females were found to be more vulnerable to these effects. In the future, professional guidance of the content on online media and weight and fitness management apps is crucial for the prevention of eating disorders in young adults.

## Background

Social environmental factors are believed to be essential risk factors for the development of disordered eating symptoms, or even eating disorders [1], a set of highly disabling and fatal mental disorders that mainly affect young females [2–4]. Among these factors, social media seem to play a very important role [5]. Since the research conducted by Becker et al. in Fiji investigating the impact of television’s introduction on the eating attitudes and behaviors in local girls [6–8], the association between media and disordered eating symptoms and its mechanism has been long explored.

Along with the rise of the Internet and smartphones, the promotion and development of online media, or new media have entered an unprecedented speed, which seems to have led to a further expansion of the media’s influence on eating disorders. Online media include online magazines, online programs, online feeds, and social media. Compared to traditional media like television or newspaper, they involve much more interactive activities, thus attracting more attention from the consumer. This would greatly increase individuals’ chance of media exposure than before [5]. The idea about the “ideal body” disseminated by the online media may be internalized by adolescents and young adults, leading them to be dissatisfied with their own bodies [9–11]. Social media like Google, Twitter, and Facebook are a part of new media. In previous studies, researchers have found that the use of social media is correlated with increased eating concern and body dissatisfaction [12–15]. Online media was also found to further increase disordered eating behaviors [16]. Notably, although some studies were mainly interested in female samples [13, 14, 17, 18], males seemed to be equally sensitive to online media [13, 15, 19]. However, males and females differed in Internet use patterns [12], and eating disorder patients of different sexes tend to have different symptom manifestations [20]. Chen et al. reported adolescent females perceived more appearance pressure from mass media in a sample from Chongqing, China [21]. So it is still necessary to investigate the relationship between online media and disordered eating symptoms separately by sex.

Another aspect that cannot be overlooked following the spread of the Internet is the emergence of various health applications (apps), especially weight and fitness management apps. The main functions of these apps are diet monitoring and calorie tracking. According to the online survey, weight and fitness management apps are among the top downloads in app stores and are prevalent among young people [22, 23]. However, this kind of “health” app does not always bring health. A previous study suggested that although young people may learn about a healthier lifestyle through these apps, they could also develop narrow and normative understandings about health [24]. For example, linking being not fat and being slim with health, and adjusting their calorie intake and exercise behaviors [24]. Some studies suggested that weight and fitness management apps with calorie tracking functions could lead to negative emotions, and even disordered eating symptoms [25–28].

Two studies investigated the negative influence of mass media from Chinese/Asian and Western on body image in Chinese young females [17, 18]. However, studies on the correlation between the use of online media and apps and disordered eating symptoms in the mainland of China are still very limited. The possible reasons are that 10 to 20 years ago, eating disorders in mainland China did not attract sufficient public attention [29]. Besides, the Internet in China was not fully covered then. At the same time, Chinese people rarely use social media like Google, Facebook, and Twitter, but instead use WeChat and Weibo, which reduces comparability with studies in other countries. Previous studies also reported different effects of Chinese and Western media on Chinese young females [17, 18]. Up to 2020, the Internet coverage rate in mainland China has reached 70.4% [30], and health apps are also emerging in large numbers under this circumstance [31]. Simultaneously, the prevalence of eating disorders in mainland China is increasing at an alarming rate [32], and China has become one of the three countries with the fastest increase in the burden of eating disorders [33]. Therefore, whether the relationship between the two is the same as that of the Western and other countries needs to be verified by research. Moreover, some studies believed that media led to disordered eating behavior through direct imitation and social learning [8, 34], while it is also reported that disordered cognition including body dissatisfaction indirectly mediated the relationship between online media and disordered eating behaviors [35, 36]. It is essential to clarify the definitive mechanism for future prevention and intervention.

The current study attempted to fill these gaps with a sample consisting of young adult college students. Accordingly, this study had two specific aims. First, to investigate whether online media exposure and weight and fitness management app use relate to disordered eating symptoms in young adults in the mainland of China. Note the online media in the current study only refers to media that would offer body shape, weight loss, and fitness-related information. Second, to further explore the mechanism through which online media and weight and fitness management app use induces disordered eating behaviors, i.e., whether online media or weight and fitness management app use directly causes disordered eating behaviors, or through evoking disordered eating cognition. We hypothesized that in mainland China, online media exposure and weight and fitness management app use correlates to disordered eating symptoms. Furthermore, we hypothesized that online media and weight and fitness management app use induce disordered eating behaviors through raising disordered eating cognition. In another word, disordered eating cognition was assumed to mediate the relationship between online media exposure, weight and fitness management app use, and disordered eating behaviors. Lastly, we hypothesized that young female adults were easier to be affected by online media or weight and fitness management apps and develop disordered eating symptoms.

## Methods

### Participants and procedure

The study was conducted between April and May 2019. Convenience sampling was adopted and the participants recruited in this cross-sectional study were freshmen in a local normal university in Shanghai, China. Students could express their willingness to participate in the study through a registration form distributed by on-campus posters and their online version. The questionnaires used in this study were also made into an online version and distributed to the participants through QR codes. The questionnaires were completed with participants’ own digital devices under the supervision of researchers in their classrooms. Each participant was assured they were free to refuse, ask questions, and discontinue at any time. A total of 615 participants attended our study and finished the questionnaires. To control the quality of the online collected data, we added three extra items at the beginning, middle, and end portions of the questionnaires. The items required the participants to select a specific option. Participants who selected the wrong options were considered not seriously filling the questionnaire, and only the questionnaires of participants who correctly answered all three items were identified as high quality and included in the final analysis. This method is generally used to detect respondents who were not paying enough attention or not obeying the questionnaire instructions [37]. We excluded participants who completed the questionnaire in low quality and were younger than 18, resulting in a final sample with 495 participants, including 142 males (28.7%) and 353 females (71.3%). The age for male participants was 19.4 2 ± 1.05, and for female participants was 19.10 ± 0.76. The participants were mainly ethnic Han Chinese (*N* = 475, 95.8%). All participants had access to the Internet through smartphones and computers and were born and raised in mainland China. The ethics committee of Shanghai Mental Health Center approved this study, and oral and written informed consent was obtained from all participants before the questionnaire was distributed.

### Measures

#### Eating Disorder Examination-Questionnaire 6.0 (EDE-Q 6.0)

Eating Disorder Examination-Questionnaire 6.0 (EDE-Q 6.0) is a widely used 28-item self-report tool evaluating disordered eating symptoms in the last 28 days [38]. The 22 items of the questionnaire make up four subscales: Dietary Restraint (5 items), Eating Concern (5 items), Shape Concern (8 items), and Weight Concern (5 items), where Shape Concern and Weight Concern share one same item. The Dietary Restraint subscale measures the disordered attitude to restrictive eating. The Eating Concern subscale investigates preoccupied cognition about food and eating. The Shape Concern subscale and Weight Concern subscale measure the dissatisfaction with body shape and body weight, respectively. The scores of the items range from 0 to 6, and higher scores represent more severe disordered eating symptoms. The subscale scores of EDE-Q were computed by averaging the corresponding items. The global score of the EDE-Q is calculated by averaging the scores of the four subscales, measuring the global severity of disordered eating symptoms. An additional six items measure disordered eating behaviors, including binge eating, self-induced vomiting, laxative misuse, and excessive exercise. The EDE-Q has been translated into Chinese and has shown good reliability and validity among the Chinese population [39]. In this study, the global score of EDE-Q was used to measure disordered eating cognition. Disordered eating behaviors were measured in five aspects, including Dietary Restriction (EDE-Q item 2), Objective Binge Episodes (EDE-Q item 14), Self-induced vomiting (EDE-Q item 16), Laxative Misuse (EDE-Q item 17), and Excessive Exercise (EDE-Q item 18). This method is consistent with previous studies [16, 40]. The EDE-Q in the current study showed relatively high internal consistency. For males, females, and the total sample, the Cronbach’s coefficient α = 0.925, 0.928, and 0.926 for the global score; α = 0.848, 0.816, and 0.826 for Eating Restraint subscale; α = 0.535, 0.605, and 0.593 for Eating Concern subscale; α = 0.868, 0.867, and 0.869 for Shape Concern subscale; and α = 0.803, 0.797, and 0.801 for Weight Concern subscale.

#### Online media exposure and weight and fitness management app use

To investigate the participants’ degree of exposure to apparent, body, and health-related information on online media, and the frequency of weight and fitness management app use, we designed a 7-item questionnaire measuring daily time spent online; frequency of receiving appearance or fitness-related information online; appearance or fitness online information seeking; percentage of time accessing appearance or fitness online information; frequency of using health software of apps with calorie calculating, tracking and weight loss functions; frequency of regulating diet according to those apps; and frequency of regulating the amount of exercise according to those apps. The full questionnaire is provided in Additional file 1: Table S1. For short, the seven items are called “Daily Internet Use”, “Media Info Receive”, “Media Info seeking”, “Media Info Proportion”, App Use”, “Diet Regulation”, and “Exercise Regulation” in methods and results.

### Statistical analysis

All statistical analyses were conducted with R software, version 4.1.0 [41]. The descriptive statistics and comparisons of demographic data, online media exposure, weight and fitness management app use, and disordered eating cognition and behaviors were performed between males and females. According to the normality examined by the Shapiro–Wilk test, the descriptive statistics of continuous variables were presented as* Mean* ± *SD* or* Median* [25th percentile (*Q1*); 75th percentile (*Q3*)], and the comparison was done with Student’s t-test or nonparametric Mann–Whitney test, respectively. With regard to sex difference tests for categorical variables, the Chi-square test was used.

To investigate the possible relationships between online media information exposure or weight and fitness management app use-related behaviors and eating disorder symptoms, Spearman’s correlation analysis was conducted. This analysis was also conducted separately by sex. To control for false-positive errors, the Bonferroni correction was used for all tests (In this study, seven online media exposure and weight and fitness management app use related items, and ten disordered eating cognition and behavior related items were included in the correlation analysis, thus the *p* values were corrected for 70 tests).

**Fig. 1:**
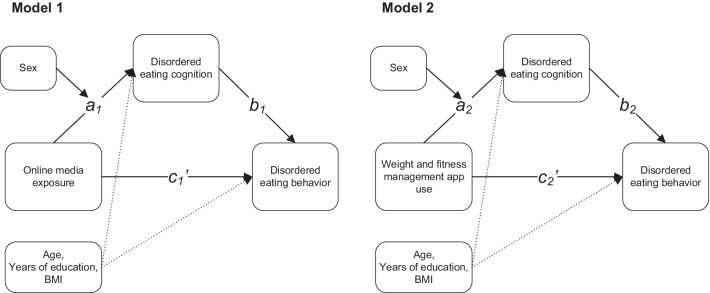
The proposed two moderated mediation models

To explore whether online media exposure or weight and fitness management apps induce disordered eating behaviors through evoking disordered eating cognition, and how sex factor affects this process, two moderated mediation models were constructed with the *bruceR* R package. The global score of EDE-Q was used to represent disordered eating cognition. The summed number of episodes of Dietary Restriction, Objective Binge Episodes, Self-induced vomiting, Laxative Misuse, and Excessive Exercise was used to represent the severity of disordered eating behaviors. The summed scores of Media Info Receive, Media Info Seeking, and Media Info Proportion were used to represent the degree of online media exposure. The summed scores of App Use, Diet Regulation, and Exercise Regulation presented the levels of involvement in weight and fitness management app use behaviors. The tested models are presented in Fig. 1. Both models take sex as the moderator, disordered eating cognition as the mediator, and disordered eating behaviors as the dependent variable. In model one, online media exposure was set as the independent variable. While in model two, weight and fitness management app use was set as the independent variable. Since the relationship between disordered eating cognition and behaviors has been well illustrated in previous studies [42, 43], we were mainly interested in the effect of the sex factor on the relationship between online media exposure or weight and fitness management apps and disordered eating cognition (path *a*_*1*_ and path *a*_*2*_), i.e., whether males and females were different in vulnerability to develop disordered eating cognition when facing these media or apps [44]. For each model, the direct effect, indirect effect, and total effect were estimated. The percentile confidence intervals of all the effects and regression coefficients were calculated through a bootstrap procedure with 1000 iterations. We included age, years of education, and BMI as covariates in the two models to rule out potential bias.

## Results

### Descriptive statistics and comparisons between sexes

The descriptive statistics and comparisons of demographic data, EDE-Q scores, number of disordered eating behavior episodes, online media exposure, and weight and fitness management app use behaviors between sexes are presented in Tables 1 and 2. The age of male participants was slightly higher than females (*p* = 0.002). The BMI of male participants was higher than females (*p* < 0.001). In terms of disordered eating symptoms, females showed significantly higher scores in Eating Concern, Weight Concern, Shape Concern, and global EDE-Q score (*p* < 0.001). With regard to online media exposure, female participants spent more daily time on the Internet (Daily Internet use *p* = 0.004), received more fashion and body-related information through online media (Media Info Received *p* < 0.001), and were more likely to look for this information through online media (Media Info Proportion *p* < 0.001). We also found female participants were more likely to use weight and fitness management apps to calculate calories and lose weight more often (App Use *p* = 0.004) and were more likely to regulate their exercise plan according to the recommendation from weight and fitness management apps (Exercise Regulation *p* = 0.016). Both male and female participants had a relatively small number of disordered eating behaviors episodes. But female participants had a higher number of episodes of objective binge eating (Objective Binge *p* < 0.001).

**Table 1:** The descriptive statistic and sex difference of demographic information, EDE-Q scores, and disordered eating behaviors

	Male (*N* = 142)		Female (*N* = 353)		*z*	*p*
	*Median*	[*Q1*;* Q3*]	*Mean*	[*Q1*;* Q3*]		
Age	19.0	[19.0;20.0]	19.0	[19.0;20.0]	3.155	0.002**
Education years	13.0	[12.0;13.0]	13.0	[12.0;13.0]	0.551	0.582
BMI	21.7	[19.5;24.6]	20.2	[18.7;21.9]	5.280	< 0.001***
EDE-Q global	0.69	[0.22;1.88]	1.22	[0.62;2.13]	− 3.750	< 0.001***
EDE-Q dietary restraint	0.60	[0.00;1.75]	0.60	[0.20;1.40]	− 1.329	0.184
EDE-Q eating concern	0.20	[0.00;0.60]	0.40	[0.20;1.00]	− 3.736	< 0.001***
EDE-Q weight concern	1.00	[0.00;2.40]	1.80	[0.80;3.00]	− 4.282	< 0.001***
EDE-Q shape concern	1.06	[0.38;2.62]	1.88	[0.88;3.25]	− 4.350	< 0.001***
Dietary restriction	0.00	[0.00;1.00]	0.00	[0.00;1.00]	− 0.477	0.633
Objective binge	0.00	[0.00;0.00]	0.00	[0.00;2.00]	− 3.585	< 0.001***
Self-induced vomiting	0.00	[0.00;0.00]	0.00	[0.00;0.00]	− 0.631	0.528
Laxative misuse	0.00	[0.00;0.00]	0.00ara>	[0.00;0.00]	− 0.870	0.385
Excessive exercise	0.00	[0.00;2.75]	0.00	[0.00;2.00]	− 0.257	0.797

**p < .01. ***p < .001

**Table 2:** The descriptive statistic and sex difference of online media exposure and weight and fitness management app use

	*N (%)*					*z*	*p*
	0	1	2	3	4		
*Daily internet use*							
Male	2 (1.4)	12 (8.5)	20 (14.1)	28 (19.7)	80 (56.3)	− 2.874	0.004**
Female	1 (0.3)	11 (3.1)	35 (9.9)	66 (18.7)	240 (68)		
*Media info receive*							
Male	23 (16.2)	56 (39.4)	38 (26.8)	12 (8.5)	13 (9.2)	− 3.484	< 0.001***
Female	30 (8.5)	99 (28)	139 (39.4)	53 (15.0)	32 (9.1)		
*Media info seeking*							
Male	46 (32.4)	37 (26.1)	46 (32.4)	7 (4.9)	6 (4.2)	− 4.551	< 0.001***
Female	43 (12.2)	107 (30.3)	137 (38.8)	38 (10.8)	28 (7.9)		
*Media info proportion*							
Male	102 (71.8)	29 (20.4)	0 (0)	10 (7.0)	1 (0.7)	− 3.814	< 0.001***
Female	188 (53.3)	114 (32.3)	34 (9.6)	11 (3.1)	6 (1.7)		
*App use*							
Male	92 (64.8)	32 (22.5)	10 (7.0)	3 (2.1)	5 (3.5)	− 2.848	0.004**
Female	184 (52.1)	82 (23.2)	63 (17.8)	10 (2.8)	14 (4.0)		
*Diet regulation*							
Male	88 (62.0)	29 (20.4)	17 (12.0)	4 (2.8)	4 (2.8)	− 1.723	0.085
Female	192 (54.4)	74 (21.0)	59 (16.7)	17 (4.8)	11 (3.1)		
*Exercise regulation*							
Male	77 (54.2)	25 (17.6)	27 (19.0)	6 (4.2)	7 (4.9)	− 2.414	0.016*
Female	151 (42.8)	71 (20.1)	77 (21.8)	32 (9.1)	22 (6.2)		

*p < .05. **p < .01. ***p < .001

### Correlation analysis

**Fig. 2:**
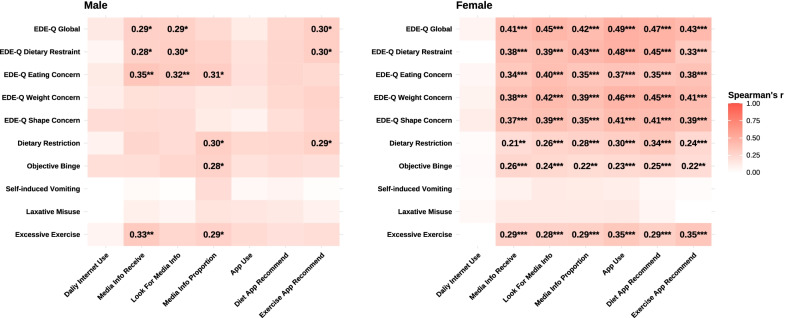
The heat map of Spearman’s correlation between online media exposure, weight and fitness management app use, and disordered eating symptoms. Note: Asterisks denote significance after Bonferroni correction. Only significant coefficients are presented

The Spearman’s correlation coefficients and their significance have been summarized in Table 3 and Fig. 2. For male participants, frequencies of passively receiving and actively looking for fashion and body shape-related information were positively correlated to disordered eating cognition about dietary restriction and eating concern. Passively receiving such information through online media was also positively associated with excessive exercising. Increased online time proportion of this information occupied was also correlated to eating concern and disordered eating behaviors like dietary restriction, objective binge, and excessive exercising. Besides, trying to achieve the recommended amount of exercise from apps was correlated to dietary restriction-related cognition and behaviors in male participants. By contrast, for female participants, except for the daily time used on the Internet, all other terms about online media exposure and weight and fitness management app use had significant linear correlations with all disordered eating symptoms. Moreover, online media exposure and weight and fitness management app use were also associated with frequencies of dietary restriction, objective binge, and excessive exercise. For detailed data distribution, see Additional file 1: Fig. S1.

**Table 3:** The Spearman’s correlation coefficients between online media exposure, weight and fitness management app use, and disordered eating symptoms

	Daily internet use	Media info receive	Look for media info	Media info proportion	App use	Diet app recommend	Exercise app recommend
*EDE-Q global*							
Male	0.14	0.29*	0.29*	0.23	0.12	0.25	0.30*
Female	0.07	0.41***	0.45***	0.42***	0.49***	0.47***	0.43***
*EDE-Q dietary restraint*							
Male	0.06	0.28*	0.30*	0.27	0.18	0.25	0.30*
Female	− 0.01	0.38***	0.39***	0.43***	0.48***	0.45***	0.33***
*EDE-Q eating concern*							
Male	0.13	0.35**	0.32**	0.31*	0.19	0.26	0.23
Female	0.05	0.34***	0.40***	0.35***	0.37***	0.35***	0.38***
*EDE-Q weight concern*							
Male	0.11	0.19	0.19	0.15	0.15	0.24	0.27
Female	0.08	0.38***	0.42***	0.39***	0.46***	0.45***	0.41***
*EDE-Q shape concern*							
Male ara>	0.22	0.22	0.22	0.12	0.08	0.20	0.26
Female	0.11	0.37***	0.39***	0.35***	0.41***	0.41***	0.39***
*Dietary restriction*							
Male	0.08	0.26	0.21	0.30*	0.25	0.25	0.29*
Female	0.03	0.21**	0.26***	0.28***	0.30***	0.34***	0.24***
*Objective binge*							
Male	0.20	0.21	0.25	0.28*	0.18	0.21	0.2
Female	0.03	0.26***	0.24***	0.22**	0.23***	0.25***	0.22**
*Self-induced vomiting*							
Male	− 0.07	0.03	0.01	0.21	0.05	0.07	0.02
Female	0.02	0.08	0.13	0.12	0.11	0.05	0.02
*Laxative misuse*							
Male	− 0.05	0.09	0.06	0.18	0.16	0.14	0.09
Female	0.05	0.13	0.13	0.13	0.13	0.07	0.01
*Excessive exercise*							
Male	0.07	0.33**	0.25	0.29*	0.22	0.19	0.20
Female	− 0.03	0.29***	0.28***	0.29***	0.35***	0.29***	0.35***

*p < .05. **p < .01. ***p < .001, Bonferroni corrected

### Moderated mediation analysis

#### Preliminary analysis

The means and pair-wise Pearson’s correlation among the variables used in the moderated mediation models are presented in Additional file 1:Table S2. The level of online media exposure, weight and fitness management app use, disordered eating cognition, and disordered eating behaviors were significantly correlated to each other.

#### Mediation effect analysis

Before testing the moderated mediation models described in Fig. 1, we first investigated the mediation effect of disordered eating cognition in the two models in the whole dataset, without sex as the moderator. The result indicated that disordered eating cognition mediated the relationship between the level of online media exposure and disordered eating behaviors (indirect effect = 1.060, *SE* = 0.152, 95% *CI* = [0.681, 1.283]). Besides, the direct effect of online media exposure on disordered eating behaviors was non-significant (direct effect = 0.301, *SE* = 0.198, 95% *CI* = [− 0.076, 0.686]), indicating a full mediation effect. Furthermore, the disordered eating cognition also mediated the relationship between weight and fitness management app use and disordered eating behaviors (indirect effect = 0.967, *SE* = 0.151, 95% *CI* = [0.682, 1.263]), and presented a full mediation effect (direct effect = 0.301, *SE* = 0.192, 95% *CI* = [− 0.071, 0.673]). The regression results for these two mediation models are presented in Additional file 1: Table S3 and S4.

#### Moderated mediation analysis

**Fig. 3:**
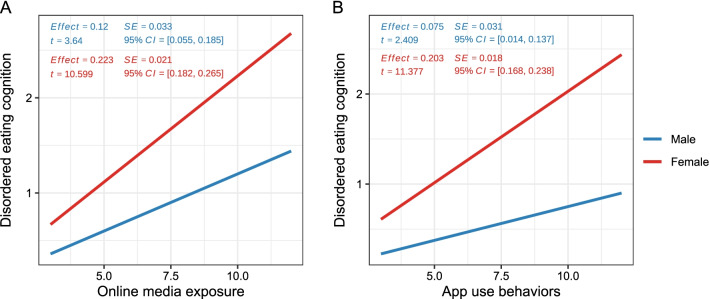
The conditional effects of sex on the relationship between (A) online media exposure or (B) app use behaviors and disordered eating cognition.* Note*:* SE* standard error;* CI* confidence interval

In the moderated mediation analysis, we further investigated whether the mediation effect of disordered eating cognition reported above was moderated by the sex factor. Two moderated mediation models were tested (see Fig. 1). The results suggested that sex significantly moderated the path *a*_1_ in model 1 (unstandardized interaction *B* =  − 0.05, *Bse* = 0.02, *t* =  − 2.634, *p* = 0.009, 95% *CI* = [− 0.10, − 0.01]) and the path *a*_*2*_ (unstandardized interaction *B* =  − 0.06, *Bse* = 0.018, *t* =  − 3.567, *p* < 0.001, *CI* = [− 0.10, − 0.02]). These results suggested that the effect of online media exposure or weight and fitness management app use on disordered eating cognition was different between males and females, and thus indicated differences in mediation effect of disordered eating cognition in the two models [44]. The regression results for the two moderated mediation models are presented in Tables 4 and 5. To clearly illustrate the moderating role of sex, simple slope tests for the two models were conducted. The results showed that compared to males, females have a larger effect of online media exposure or weight and fitness management app use on disordered eating cognition (see Fig. 3). In both models showed in Fig. 1, females showed a larger mediation effect (i.e., indirect effect) of disordered eating cognition (model 1: indirect effect of males = 0.642, *SE* = 0.214, 95% *CI* = [0.215, 1.031]; indirect effect of females = 1.191, *SE* = 0.203, 95% *CI* = [0.854, 1.663]; model 2: indirect effect of males = 0.417, *SE* = 0.188, 95% *CI* = [0.048, 0.787]; indirect effect of females = 1.120, *SE* = 0.168, 95% *CI* = [0.794, 1.450]).

**Table 4:** The moderated mediation effect of disordered eating cognition on the relationship between online media exposure and disordered eating behaviors

Predictors	(Regression 1) DE behaviors	(Regression 2) DE cognition	(Regression 3) DE behaviors
(Intercept)	5.626***	1.258***	6.252***
	(0.427)	(0.046)	(0.417)
Age	0.438	− 0.014	0.396
	(0.497)	(0.047)	(0.437)
Education years	− 0.016	0.000	− 0.012
	(0.188)	(0.017)	(0.164)
BMI	0.235*	0.099***	− 0.336**
	(0.118)	(0.011)	(0.115)
Online media exposure	1.558***	0.172***	0.578**
	(0.188)	(0.020)	(0.187)
Sex		− 0.177***	1.467**
		(0.048)	(0.446)
Online media exposure × Sex		− 0.051**	
		**(0.020)**	
DE cognition			5.334***
			(0.423)
*R*^2^	0.131	0.327	0.347
Adj. *R*^2^	0.124	0.319	0.339
Num. obs	495	495	495

Unstandardized regression coefficients are displayed, with standard errors in parentheses

DE denotes disordered eating

*p < .05. **p < .01. ***p < .001

**Table 5:** The moderated mediation effect of disordered eating cognition on the relationship between app use behaviors and disordered eating behaviors

Predictors	(Regression 1) DE behaviors	(Regression 2) DE cognition	(Regression 3) DE behaviors
(Intercept)	5.626***	1.249***	6.198***
	(0.432)	(0.045)	(0.419)
Age	0.331	− 0.015	0.362
	(0.502)	(0.046)	(0.440)
Education years	− 0.006	0.003	− 0.002
	(0.191)	(0.017)	(0.165)
BMI	0.127	0.089***	− 0.371**
	(0.121)	(0.011)	(0.115)
App use behaviors	1.268***	0.139***	0.332*
	(0.170)	(0.018)	(0.166)
sex		− 0.209***	1.342**
		(0.047)	(0.446)
App use behaviors × sex		− 0.064***	
		**(0.018)**	
DE cognition			5.532***
			(0.426)
*R*^2^	0.110	0.337	0.340
Adj. *R*^2^	0.103	0.329	0.331
Num. obs	495	495	495

Unstandardized regression coefficients are displayed, with standard errors in parentheses

For short, weight and fitness management app use behaviors are described as app use behaviors, and DE denotes disordered eating

*p < .05. **p < .01. ***p < .001

## Discussion

In the current study, we obtained three main results. First, we detected essential differences in disordered eating symptoms, online media exposure, and weight and fitness management app use patterns between sexes. In general, females manifested more severe disordered eating cognition and behaviors and were more likely to be passively or actively exposed to body shape and appearance-related information online. Females were also more likely to use weight and fitness management apps and follow the recommendations from them. Second, a strong correlation between online media exposure, weight and fitness management app use and disordered eating symptoms was found, especially in females. Third, we observed that disordered eating cognition mediated the relationship between online media exposure and weight and fitness management app use and disordered eating behaviors, and this effect is significantly moderated by the sex factor.

Consistent with previous studies in Western and Chinese samples [20, 45], we found that the prevalence and severity of disordered eating symptoms were higher in females than males among young adults. The difference between sexes in online media use patterns also exhibited cross-cultural consistency. In an investigation conducted in Taiwan, it was reported that females had higher levels of thin-ideal media exposure [46]. Murray et al. reported that in a sample of Ottawa, young female adults tended to spend more time on social networking sites than males [36]. In a cross-sectional survey with a large sample of American young adults, it was also reported that young female adults spent significantly more time every day on social media than males [15]. In the background of thinness as beauty, females were believed to perceive higher peer pressure and body dissatisfaction [47], and were passively or actively exposed to the body shape-related online information, and then began to use health-related apps.

Through correlation analysis, we found that in both male and female young adults, online media exposure was associated with disordered eating cognition and behaviors, but such associations were less broad in young male adults. Importantly, the results of the current study suggested that the influence of the Internet on disordered eating symptoms is mainly attributed to its contents conveyed by media, rather than the time spent on it, for both males and females. The inappropriate information about body shape and weight distributed by online media including social media may contribute to disordered eating cognition and behaviors. As suggested by Michels et al., reducing body size dissatisfaction and improving body esteem may be an appropriate way to prevent disordered eating symptoms [9].

It is worth noting that the use of weight and fitness management apps to calculate calories, regulate diet and exercise may significantly increase disordered eating cognition, dietary restriction, objective binge eating, and excessive exercise in females. For males, trying to achieve the recommended amount of exercise from apps was also correlated to dietary restriction-related cognition and behaviors. As a product of the rapidly growing Internet, weight and fitness management apps with calorie and weight monitoring functions are getting popular among young adults [48]. Several studies have reported the association between these apps and disordered eating cognition and behaviors in college students, especially in dietary restriction and excessive exercise [27, 28]. The conclusion is highly consistent across these studies that using these apps is significantly correlated with increased disordered eating cognition and behaviors like compulsive exercise. Levinson et al. also found that these apps users accounted for a considerable proportion of patients with eating disorders [25], which may reveal the mutual promotion effect between app use and disordered eating. The current study provided supplementary evidence to these conclusions in a wider population.

Through the mediation analysis, we found that online media exposure and weight and fitness management app use indirectly induced disordered eating behaviors through evoking disordered eating cognition, in both males and females. Several prior studies have investigated the mechanisms of how disordered eating symptoms were caused by media exposure. Murray et al. reported that appearance and weight esteem fully mediated the relationship between time spent on social networking sites and restraint eating [36]. Fortes et al. also found that body dissatisfaction mediated the relationship between media pressure and disordered eating behaviors [35]. The internalization of the values conveyed by online media and weight and fitness management apps may raise disordered eating cognition, which is an essential factor causing disordered eating behaviors[10, 46], and should be treated as a crucial point for future prevention and intervention of eating disorders. This indicated that under such a media-covered society, intervention on disordered eating cognition is equally, or even more important than correcting disordered eating behaviors. Considering the limited social attention to eating disorders in mainland China [29], some actions should be taken to prevent negative consequences that may be caused by online media. For instance, encouraging or enforcing social responsibility of online media platforms and weight and fitness management apps, and promoting diversity or alternative attitudes about weight and appearance within society through values guidance in education. Besides, as Goodyear et al. recommended, the researchers and policymakers are responsible to strengthen the evidence base on process in which young people use and navigate digital health technologies [24].

Some systematic reviews reported that sex may not modulate the relationship between media and disordered eating [13, 49]. Though in the current study, the correlation analysis and moderated mediation analysis indicated that young males could be affected by online media exposure or weight and fitness management app use like females, the extent of the effect seemed substantially different. For young adult females, almost all online media exposure and weight and fitness management app use related behaviors associated with disordered eating cognition, while such connections were less broad in young adult males. The moderated mediation analysis further suggested that compared to males, females were more vulnerable to the effect of online media and weight and fitness management apps, and were more likely to raise disordered eating cognition when facing them, which was consistent with a previous study in a Chinese adolescent sample [21]. This may be attributed to the different appearance expectations from society for males and females in the mainland of China. Females are usually facing more appearance demand than males. McCabe et al. believed that females could be aware of these expectations at an early age. So they were more likely to adopt harmful strategies to achieve the expectations [50]. While for males, appearance seemed less important and thus they perceive less pressure from online media and apps. Differences in culture, online platforms, and the interested disordered eating cognition and behaviors in various studies may also lead to inconsistency across studies.

The current study investigated media exposure, weight and fitness management app use, and their influence on disordered eating symptoms in Chinese mainland young adults. Furthermore, this study benefited from a strict quality control procedure, which enhanced its reliability. However, some limitations should be acknowledged. First, we adopted a cross-sectional design, which restricted its ability to draw causal inferences. Though we hypothesized that it was online media and apps that induced disordered eating symptoms, some evidence also suggested that body dissatisfaction and personality also affect weight and fitness management app use [51, 52]. Second, our sample was restricted to a small age range, which prevented our conclusion from generalizing to a larger population. Third, the detailed behaviors when participants surf the Internet were not investigated. More thorough and detailed studies are needed in the future.

In conclusion, more attention should be paid to the high sensitivity of young adults to the online media effect, especially females, and scientific guidance is recommended to the existing content from online media and apps. Furthermore, the importance of intervention on disordered eating cognition should be emphasized. Besides, the advantages of the Internet including online intervention and disease detection with big data techniques should also be noticed [53–55], which may help exert the positive effect of the Internet on the prevention and intervention of eating disorders.

## Supplementary Information

**Additional file 1**.** Fig. S1 and Table S1–S4**. Data distribution; questionnaire items; results of preliminary correlation analysis and mediation analysis.

## Data Availability

The datasets used and/or analyzed during the current study are available from the corresponding author on reasonable request.
