# Supplementary material for: Online media exposure and weight and fitness management app use correlate with disordered eating symptoms: evidence from the mainland of China
Source: J Eat Disord. 2022 Apr 25;10:58. doi: 10.1186/s40337-022-00577-y (PMC9036716; doi:10.1186/s40337-022-00577-y)
Supplement: Supplementary file 1 — Additional file 1. Fig. S1 and Table S1–S4. Data distribution; questionnaire items; results of preliminary correlation analysis and mediation analysis. [file 40337_2022_577_MOESM1_ESM.docx]

**
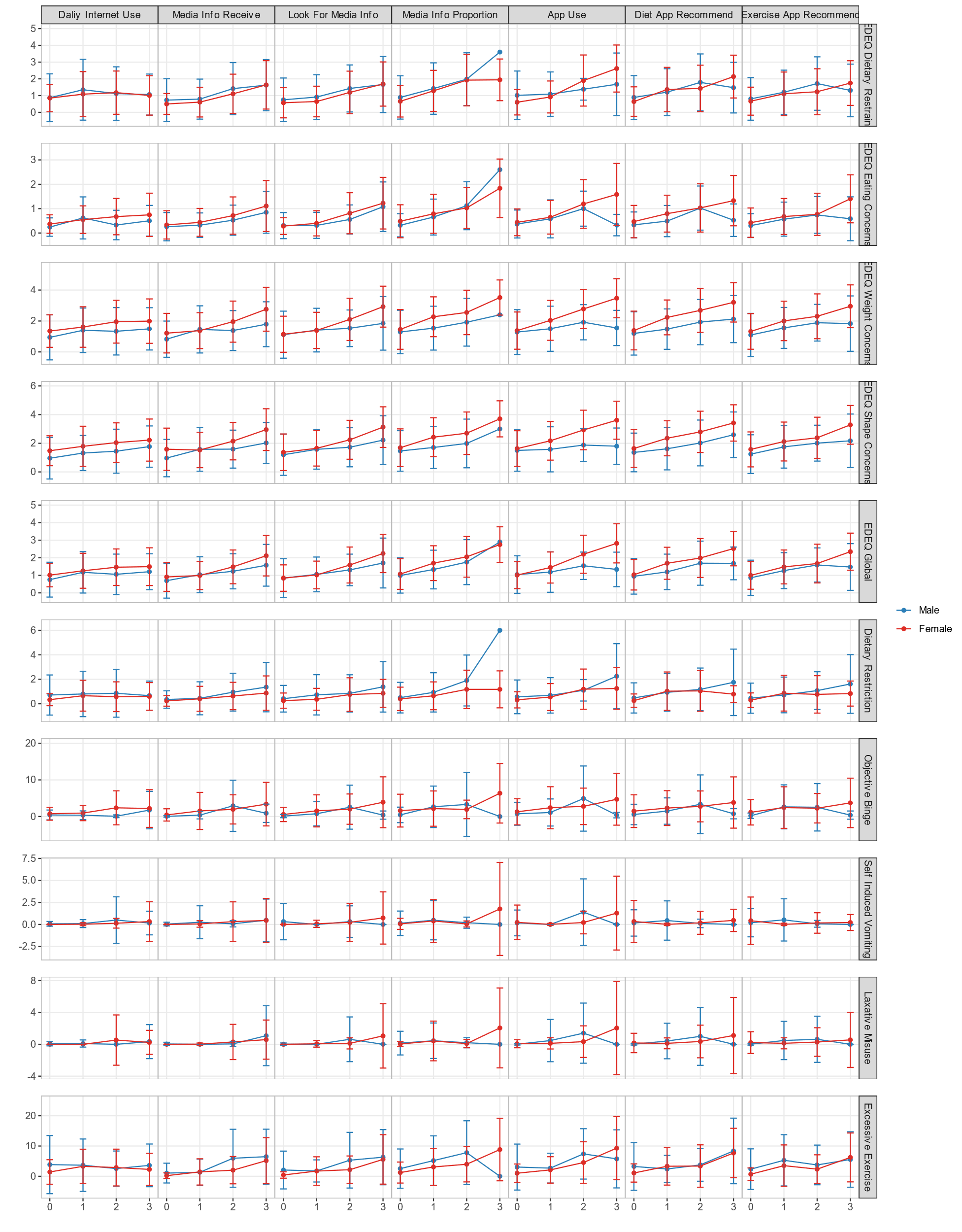
**

**Fig. S1** The distribution of disordered eating symptoms based on online media exposure or figure management app use items in males and females. Each node represents the mean value of the EDE-Q subscale or total score at the corresponding level of online media exposure or health app use. The error bars represent the standard deviation. The lines in blue represent males while the lines in red represent females.

**Table S1** The questionnaire used to measure online media exposure and weight and fitness management app use

| Score | 0 | 1 | 2 | 3 |
| --- | --- | --- | --- | --- |
| 1. How long do you spend on the Internet every day on average (including with smartphones)? | 0~2hr | 2~3hr | 3~4hr | > 4hr |
| 2. When you are online, how often do you receive information about fashion, weight loss, fitness, body shape, or appearance, etc.? | never | seldom | sometimes | often |
| 3. When you are online, how often do you actively look for such information described above | never | seldom | sometimes | often |
| 4. What is the percentage of the time you spent on such information accounting for your total online time | 0~10% | 10~20% | 30~40% | >40% |
| 5. How often do you use health software or apps for calorie calculating, tracking or weight loss? | never | seldom | sometimes | often |
| 6. How often do you regulate your diet according to the calorie intake recommended in those apps (even without success)? | never | seldom | sometimes | often |
| 7. How often do you try to achieve the recommended amount of exercise in the apps every day (even without success) | never | seldom | sometimes | often |

| **Variables** | ***Mean± SD*** | **Age** | **Education years** | **BMI** | **Online media exposure** | **App use behaviors** | **DE cognition** | **DE behaviors** |
| --- | --- | --- | --- | --- | --- | --- | --- | --- |
| **Age** | 19.19± 0.87 | - |  |  |  |  |  |  |
| **Education years** | 12.18± 2.28 | 0.06 | - |  |  |  |  |  |
| **BMI** | 21.34± 3.62 | 0.01 | -0.05 | - |  |  |  |  |
| **Online media exposure** | 6.78± 2.29 | -0.10* | 0.05 | 0.04 | - |  |  |  |
| **App use behaviors** | 5.47± 2.59 | -0.08 | 0.04 | 0.16*** | 0.55*** | - |  |  |
| **DE cognition** | 1.36± 1.06 | -0.07 | 0.01 | 0.32*** | 0.46*** | 0.49*** | - |  |
| **DE behaviors** | 5.63± 10.16 | 0.01 | 0.01 | 0.10* | 0.35*** | 0.33*** | 0.56*** | - |

**Table S2.** Descriptive statistics and Pearson’s correlation coefficients among variables for moderated mediation analysis

*Note. N* = 495. **p* < .05. ***p* < .01. ****p* < .001.

For short, weight and fitness management app use behaviors are described as app use behaviors, and DE denotes disordered eating.

**Table S3.** The mediation effect of disordered eating cognition on the relationship between online media exposure and disordered eating behaviors

| **Predictors** | **(Regression 1) DE behaviors** | **(Regression 2) DE cognition** | **(Regression 3) DE behaviors** |
| --- | --- | --- | --- |
| **(Intercept)** | 5.626^***^ | 1.356^***^ | 5.626^***^ |
|  | (0.427) | (0.040) | (0.375) |
| **Age** | 0.438 | –0.034 | 0.612 |
|  | (0.497) | (0.047) | (0.437) |
| **Education years** | –0.016 | –0.002 | –0.008 |
|  | (0.188) | (0.018) | (0.165) |
| **BMI** | 0.235^*^ | 0.089^***^ | –0.223^*^ |
|  | (0.118) | (0.011) | (0.111) |
| **Online media exposure** | 1.558^***^ | 0.207^***^ | 0.498^**^ |
|  | (0.188) | (0.018) | (0.187) |
| **DE cognition** |  |  | 5.132^***^ |
|  |  |  | (0.422) |
| ***R*^2^** | 0.131 | 0.303 | 0.333 |
| **Adj. *R*^2^** | 0.124 | 0.297 | 0.326 |
| **Num. obs.** | 495 | 495 | 495 |

*Note*. Unstandardized regression coefficients are displayed, with standard errors in parentheses.

DE denotes disordered eating.

* *p* < .05. ** *p* < .01. *** *p* < .001.

**Table S4.** The mediation effect of disordered eating cognition on the relationship between app use behaviors and disordered eating behaviors

| **Predictors** | **(Regression 1) DE behaviors** | **(Regression 2) DE cognition** | **(Regression 3) DE behaviors** |
| --- | --- | --- | --- |
| **(Intercept)** | 5.626^***^ | 1.356^***^ | 5.626^***^ |
|  | (0.432) | (0.040) | (0.376) |
| **Age** | 0.331 | –0.045 | 0.568 |
|  | (0.502) | (0.047) | (0.438) |
| **Education years** | –0.006 | –0.001 | 0.000 |
|  | (0.191) | (0.018) | (0.166) |
| **BMI** | 0.127 | 0.073^***^ | –0.261^*^ |
|  | (0.121) | (0.011) | (0.110) |
| **App use behaviors** | 1.268^***^ | 0.182^***^ | 0.301 |
|  | (0.170) | (0.016) | (0.167) |
| **DE cognition** |  |  | 5.306^***^ |
|  |  |  | (0.423) |
| ***R*^2^** | 0.110 | 0.298 | 0.327 |
| **Adj. *R*^2^** | 0.103 | 0.292 | 0.320 |
| **Num. obs.** | 495 | 495 | 495 |

*Note*. Unstandardized regression coefficients are displayed, with standard errors in parentheses.

For short, weight and fitness management app use behaviors are described as app use behaviors.

* *p* < .05. ** *p* < .01. *** *p* < .001.
